# Editorial: Advances in craniopharyngioma: From physiology to clinical management

**DOI:** 10.3389/fneur.2023.1118806

**Published:** 2023-02-10

**Authors:** Songbai Gui

**Affiliations:** Department of Neurosurgical Oncology, Beijing Tiantan Hospital, Capital Medical University, Beijing, China

**Keywords:** craniopharyngioma, advances, physiology, clinical management, future

Craniopharyngioma (CP) is any epithelial tumor that originates from remnants of the craniopharyngeal duct epithelium. There are 0.5–2.5 new cases per million people per year worldwide. CP accounts for 1.2–4.6% of all intracranial tumors and 5–11% of brain tumors in children (1, 2). The clinical manifestations include pituitary/hypothalamic deficiencies, visual impairment, and increased intracranial pressure. These symptoms are caused by the tumor mass impacting the optic nerves/chiasm and hypothalamic–pituitary axis. In the early twentieth century, surgery for CP was extremely challenging and risky owing to the close anatomical proximity to the optic chiasm and hypothalamic–pituitary axis. Quality of life (QOL) and neuropsychological function are frequently impaired following surgery, such as visual deterioration, neuroendocrine deficiencies and hypothalamic injury (3). Current treatment strategies are debated, ranging from radical surgical strategies such as gross-total resection (GTR) and the extended transsphenoidal endoscopic endonasal approach (EEA) to limited surgical approaches focused on the preservation of hypothalamic and visual integrity and QOL after treatment (4). Therefore, research on how to protect neurological structures and functions of the optic nerve, pituitary stalk and hypothalamus during the surgical resection of CP is crucial. This Research Topic focused on the protection of important structures during surgical resection of CP, the mechanism of the development of CP and its related neurocognitive deficits. The aim of this Research Topic was to integrate the high-quality and up-to-date advances in the above fields. Therefore, we enrolled 20 articles (including 16 original research, 3 reviews and 1 opinion) covering the following themes:

1. Intraventricular craniopharyngiomas (IVCs): In the last few decades, experience gained from using the EEA has made this technique the gold standard for treating most IVCs. This group of articles studied special features in clinical presentation, imaging, management, and surgical outcome of IVCs (Deopujari et al.). Their deep-seated location and limited surgical field of-view makes minimally invasive EEA most suitable for their excision, especially the expanded Transsphenoidal Trans-Lamina Terminalis Approach (TLTA) (Cao et al.). Notably, the concept of “maximum safe resection,” which prioritizes the preservation of hypothalamic functions and psychological autonomy over the completeness of resection should guide surgical actions when dealing with such a complex lesion (Pascual et al.).
2. Pediatric craniopharyngiomas: Extended endoscopic endonasal resection of CP may be used as a safe and effective approach for children. Due to the poor pneumatization of the sphenoid sinus and Narrow surgical space in children, surgical techniques of exposing the sellar region, removing the tumor, and reconstructing the skull base, as well as post-operative management of patients were proposed (Wu D. et al.). Remarkably, Boekhoff et al. also analyzed the incidence of cerebral infarction (CI) in a cohort of 244 German childhood-onset CP patients recruited between 2007 and 2019 with a high degree of completeness in the prospective, randomized trial KRANIOPHARYNGEOM 2007.
3. Hypothalamic involvement/invasion: Hypothalamic damage may severely impair the QOL of patients and has an impact on long-term mortality. The most dramatic complication is the development of a hypothalamic syndrome (HS), which is typically associated with neuroendocrine disorders and includes neurocognitive changes, morbid hypothalamic obesity and related systemic complications, a variety of sleep disorders (Romigi et al.), and metabolic syndrome (Scarano et al.). Neurocognitive and physiopathological assessment and intervention before and after surgery are important in patients with larger tumors, invading the third ventricle, and tumors with hypothalamic invasion (Zhao R. et al.). The management of patients suffering from HS should be multidisciplinary, and future avenues for development of new drugs may hopefully lead to positive effects.
4. Visual protection: Specific to visual function protection, visual-evoked potentials (VEP) have proven to be an effective modality for reflecting the integrity of visual pathway from retina to the pulvinar cortex. Furthermore, optical coherence tomography (OCT) can serve as a non-invasive *in vivo* method to quantitatively and objectively measure thinner circumpapillary retinal nerve fiber layer (cpRNFL) and macular ganglion cell complex (mGCC), which have been applied to surgeries with the risk of visual pathway damage and serve as a predictor of visual recovery after surgery. Two studies found that OCT and VEP were valuable for predicting post-operative visual function in patients undergoing CP resection *via* extended EEA and intraoperative VEP monitoring is an effective method for preventing visual deterioration (Qiao et al.; Tao et al.).
5. Endocrinological function protection/pituitary stalk preservation: One article reviewed the latest research progress on the pathogenesis, presentation, significance, and treatment of endocrine disorders in patients with CP (Zhou et al.). And Tao Hong retrospectively studied a total of 183 patients with CP and demonstrated that intact hypothalamic structure is critical in maintaining pituitary function, suggesting that the pituitary stalk infiltrated by CP could be sacrificed to achieve radical resection, without substantially rendering significantly worse endocrinological efficiency 1 year after surgery (Wu J. et al.). Whereas, Chen et al. found that preserving the pituitary stalk does not appear to increase the risk of non-GTR and tumor recurrence/progression and might help reduce the risk of surgically induced hypothyroidism and diabetes insipidus. Thus, the latter authors recommend preserving the pituitary stalk in peripheral type suprasellar CP with normal pituitary function, especially in cases without hypothyroidism or diabetes insipidus, and stalk sacrifice can be considered in central type tumors with severe pre-operative endocrinopathy.
6. Surgical approach/strategy comparison: A retrospective review compared surgical outcomes and complications between transcranial surgery (TCS) and endoscopic endonasal surgery (EES) of CP (Nie et al.). The conclusion supported the view that EES is a safe and effective minimally invasive surgery compared to TCS. Compared to TCS, EES has fewer surgical complications and a lower recurrence rate (5).
7. Basic research: One study performed unsupervised cluster analysis on the 725 immune-related genes and arrays of 39 patients with adamantinomatous craniopharyngioma (ACP) patients in GSE60815 and GSE94349 databases. Two novel immune subtypes were identified, namely immune resistance (IR) subtype and immunogenic (IG) subtype. The expression levels of immune checkpoint molecules (PD1, PDL1, PDL2, TIM3, CTLA4, Galectin9, LAG3, and CD86) were significantly upregulated in the IG subtype (Yuan et al.). Guo introduced an integrated algorithm for identifying lncRNAs and TFs regulating the ACP-related pathway, which may serve as a valuable resource for understanding the mechanisms underlying ACP-related lncRNAs and TFs (Xu, Guo et al.).
8. New classification/technology: A WDR89-based nomogram mode was constructed to predict the immune classification of ACP with excellent performance (Yuan et al.). This predictive model provided a reliable classification assessment tool for clinicians and aids treatment decision-making in the clinic. The technical route of intelligent diagnosis is based on the application of traditional machine learning and deep learning models in the clinical diagnosis of CP from the aspects of differential classification, prediction of tissue invasion and gene mutation, prognosis prediction, and so on (Qin et al.). One study aimed to establish and validate a nomogram based on preoperative imaging features and blood indices to differentiate between cystic-solid pituitary adenomas and CP (Zhao Z. et al.).
9. Future trends: Exploring novel methods of automatized analysis of data for gaining knowledge in any medical field is an encouraging task, particularly in such an extremely challenging tumor as CP. Two articles researched the clinical features and long-term recurrence of CP, investigated the research trends and evaluated research hotspots using bibliometric analysis and nomograms of a retrospective, multiple-center, cohort study, separately (Li et al.; Xu, Wei et al.). This research provides a comprehensive analysis of the scientific progress of CP in the past decades, and insight into the development of the CP research field, highlights research trends over time, and helps identify valuable future directions, whose conclusions could serve as the practical tool for individual strategies based on the patient's baseline characteristics to achieve a better prognosis.

In conclusion, professional expertise and advanced technology in diagnostics and treatment have a relevant impact on outcome and prognosis after CP. Multicenter-based networks for reference assessments should be considered to assure high standards of treatment quality (6). Future efforts to improve prognosis, outcome and QOL in patients with CP should be focused on improving our understanding of the molecular pathogenesis of CP, with the perspective of developing targeted therapies effective against progression and hypothalamic involvement as well as surgical and radio-oncological treatment strategies, aiming at hypothalamus-sparing approaches to prevent sequelae, and treatments and interventions for hypothalamic obesity and neuropsychological sequelae after CP resection. Furthermore, policy efforts should be made to establish and confirm criteria for the quality of multidisciplinary treatment of CP as well as to improve the infrastructure of surgical instrumentation to provide equitable care across the world (7).

## Author contributions

The author confirms being the sole contributor of this work and has approved it for publication.
